# *Streptococcus intermedius* causing primary bacterial ventriculitis in a patient with severe periodontitis - a case report

**DOI:** 10.1186/s12883-024-03604-4

**Published:** 2024-04-05

**Authors:** Satu Allonen, Janne Aittoniemi, Matti Vuorialho, Lassi Närhi, Kari Panula, Risto Vuento, Jari Honkaniemi

**Affiliations:** 1 Mehiläinen, Tammisaari, Finland; 2 Department of Clinical Microbiology, Fimlab laboratories, Arvo Ylpon katu 4, 33520 Tampere, Finland; 3 Department of Radiology, Vaasa Central Hospital, Vaasa, Finland; 4 Department of Physical and Rehabilitation Medicine, Päijät-Häme Central Hospital, Lahti, Finland; 5 Department of Oral and Maxillofacial Surgery, Vaasa Central Hospital, Vaasa, Finland; 6 Department of Neurology, Vaasa Central Hospital, Vaasa, Finland; 7 Tampere University Hospital, Tampere and Turku University, Turku, Finland

**Keywords:** Bacterial infections, Hydrocephalus, MRI

## Abstract

**Background:**

*Streptococcus intermedius* is a member of the *S. anginosus* group and is part of the normal oral microbiota. It can cause pyogenic infections in various organs, primarily in the head and neck area, including brain abscesses and meningitis. However, ventriculitis due to periodontitis has not been reported previously.

**Case presentation:**

A 64-year-old male was admitted to the hospital with a headache, fever and later imbalance, blurred vision, and general slowness. Neurological examination revealed nuchal rigidity and general clumsiness. Meningitis was suspected, and the patient was treated with dexamethasone, ceftriaxone and acyclovir. A brain computer tomography (CT) scan was normal, and cerebrospinal fluid (CSF) Gram staining and bacterial cultures remained negative, so the antibacterial treatment was discontinued. Nine days after admission, the patient’s condition deteriorated. The antibacterial treatment was restarted, and a brain magnetic resonance imaging revealed ventriculitis. A subsequent CT scan showed hydrocephalus, so a ventriculostomy was performed. In CSF Gram staining, chains of gram-positive cocci were observed. Bacterial cultures remained negative, but a bacterial PCR detected *Streptococcus intermedius*. An orthopantomography revealed advanced periodontal destruction in several teeth and periapical abscesses, which were subsequently operated on. The patient was discharged in good condition after one month.

**Conclusions:**

Poor dental health can lead to life-threatening infections in the central nervous system, even in a completely healthy individual. Primary bacterial ventriculitis is a diagnostic challenge, which may result in delayed treatment and increased mortality.

## Background

Bacterial infections of the central nervous system caused by oral microbiota are uncommon in industrialized countries, with a prevalence of only 1–2%, despite the common occurrence of various dental infections. The most common bacterial infections of the brain are meningitis and cerebral abscesses, while ventriculitis is rare [1, 2]. Ventriculitis caused by oral infections has not been previously reported. Here, we present the first case of ventriculitis caused by an oral pathogen.

## Case presentation

A previously healthy 64-year-old male was admitted to the hospital with headache, fever and later imbalance, blurred vision and general slowness. Patient’s neurological examination revealed nuchal rigidity and general clumsiness. Blood tests showed leukocytosis (11.90 × 10^9^/l) and an increased C-reactive protein (47 mg/l). A computer tomography (CT) scan of the brain showed normal results. The cerebrospinal fluid (CSF) was clear but yellowish and had a slightly elevated count of erythrocytes (10 × 10^6^/l), high levels of leukocytes (940 × 10^6^/l; 40% lymphocytes and 56% granulocytes), elevated protein levels (1,696 mg/l), and hypoglycorrhachia (0.9 mmol/l). Bacterial cultures and CSF staining remained negative.

Meningitis was suspected, and the patient was treated with intravenous (IV) dexamethasone (10 mg four times a day), ceftriaxone (4 g daily), and acyclovir (750 mg three times a day). Four days after admission, when his C-reactive protein had decreased to 10 mg/l, viral meningitis was considered the most probable cause. As a result, the treatment with ceftriaxone and dexamethasone was discontinued, but the acyclovir treatment continued.

**Fig. 1:**
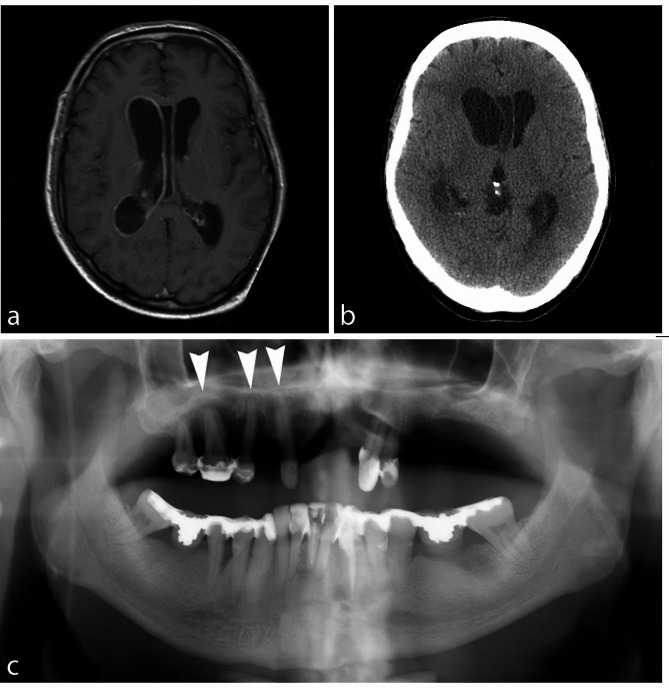
**a**. Gadolinium enhanced T1 MR image. The ependyma of the right lateral ventricle and the cavum septi pellucidi enhances intensively, as a sign of ventriculitis. Lateral ventricles are slightly enlarged. **b**. Unenhanced CT slice on the level of frontal horns. Hydrocephalus has progressed, especially on the right side, the right frontal horn has enlarged and the anterior part of cavum septi pellucidi deviates to the left. Cortical sulci are narrowed compared to the previous examination. **c**. General chronic periodontitis in both jaws and severe local bone loss with abscesses. Periapical abscess is indicated by arrow

Nine days after admission, the patient’s general condition slowly deteriorated, and he became increasingly somnolent. The patient was restarted on IV ceftriaxone (2 g daily, which was increased to 4 g daily one day later), in combination with doxycycline (100 mg twice a day) due to the suspicion of borreliosis. On the following day, brain magnetic resonance imaging (MRI) was performed, and it revealed signs compatible with ventriculitis in the right lateral ventricle and the third ventricle (Fig. 1a).

Eleven days after admission, the patient’s consciousness rapidly declined. A new CT scan of the brain revealed hydrocephalus and a mild midline shift, attributed to the enlarged right lateral ventricle (Fig. 1b), so an emergency ventriculostomy was performed. Cerebrospinal fluid obtained during the operation appeared clear but yellowish, with later debris observed in the CSF collector bag. Doxycycline was discontinued. Due to the neurosurgeon’s suspicion of a poor clinical response to ceftriaxone, it was switched to IV cefotaxime (2 g three times a day), with the dosage increased to 2 g four times a day by an infection consultant two days later. No signs of renal dysfunction were detected, and serum creatinine levels remained within the normal range. The patient’s condition rapidly improved after the ventriculostomy and antibiotic treatment. On the 21st day after admission, the ventriculostomy was closed.

In the Gram staining of the CSF sample obtained from the ventriculostomy at the time of the operation, chains of gram-positive cocci were observed inside polymorphonuclear leukocytes. The bacteria’s morphology resembled that of streptococci. Bacterial cultures of both CSF and blood remained negative. The sample, which displayed bacteria in the Gram staining, and another CSF sample taken one day later, were analyzed using in-house bacterial 16s ribosomal RNA gene amplification by polymerase chain reaction (PCR) with high sensitivity for both aerobic and anaerobic bacteria, followed by sequencing. The analysis of both samples tested positive for *S. intermedius*.

**Fig. 2:**
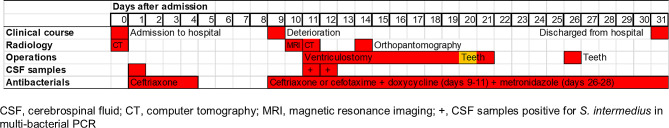
The clinical time course, and the most important examinations and interventions of the patient with *S. intermedius* ventriculitis during hospitalisation

No clinical signs of infective endocarditis were observed in further assessments, and echocardiography was not performed. The patient mentioned a history of chronic dental problems. An orthopantomography revealed advanced periodontal destruction in several teeth, and periapical abscesses were found in teeth 33 and 31 (Fig. 1c). Maxillary teeth 15 and 16 were urgently extracted, followed by the extraction of teeth 17, 23, 24, 31, 32, 33, and 43. During the latter operation, prophylactic metronidazole (500 mg three times a day) was initiated for three days. The patient continued to improve and was discharged in good condition one month after admission, with only slight left-sided hemiparesis. The clinical time course, and the most important examinations and interventions of the patient during hospitalisation are illustrated in Fig. 2.

## Discussion and conclusions

The most common predisposing factors for ventriculitis are penetrating trauma, intracranial bleeding, neurosurgical procedures, devices, meningitis, cerebral abscesses, and an immunosuppressive state [2]. The clinical features of ventriculitis resemble those of meningitis, along with altered mental status [3], and mortality varies between 30% and 70% [4]. The diagnosis of ventriculitis can be challenging, as in the present case, where bacterial cultures of CSF and blood remained negative. Bacterial culture of CSF is not very sensitive in ventriculitis, often due to prior antimicrobial therapy [5]. Fabre et al. published recently a review article in which they estimated the pretest probability of bacteremia and blood culture positivity in common clinical scenarios. In cases of meningitis, the probability is high, but lower in, for example, ventricular shunt infections [6].

If ventriculitis is suspected, MRI can confirm the diagnosis. Characteristic MRI findings include abnormal periventricular and subependymal signal intensity, enhancement of the ventricular lining, and sometimes signs of intraventricular debris and pus [7]. In the present case, the MRI findings were limited to contrast enhancement of the ventricular lining with no intraventricular debris. Nevertheless, debris was detected in the CSF obtained by ventriculostomy, and it was thought to have caused the hydrocephalus by obstructing the right foramen of Monro.

A variety of bacteria, including enterobacteria, staphylococci, and streptococci, can cause ventriculitis, depending on the source of infection, such as shunts, surgical procedures, trauma, or hematogenous spread [2, 7, 8]. Primary bacterial or pyogenic ventriculitis typically occurs in children. Only twelve cases of primary bacterial ventriculitis have been reported in adults [9–13], with one of them caused by *S. intermedius* [14]. This pathogen is known to be a member of normal oral microbiota and is a frequent cause of periodontitis and oral abscesses. As part of the *S. anginosus* group, *S. intermedius* is considered an emerging pathogen, and there are differences in pathogenicity among different strains. However, in contrast to other streptococcal pathogens, the virulence factors and their regulation in *S. intermedius* are still poorly understood [15]. To our knowledge, this is the first reported case of isolated ventriculitis as a complication of oral infection. Our patient’s case highlights the potential risks of poor dental conditions even in a completely healthy individual. If an infection is supposed to be of dental origin, the pathogen should represent the oral microflora, there should be radiographical signs of dental or paradental infection and other sources of infection must be ruled out [16]. All these criteria are met in the present case, though a culture of the periodontal abscesses was not done. In patients previously treated with antibiotics, bacterial cultures of blood and CSF often remain negative. In such situations, multiplex bacterial PCR methods may be useful in establishing a diagnosis.

## Data Availability

The dataset supporting the conclusions of this article is included within the article.

## Abbreviations

CSF: Cerebrospinal fluid
CT: Computer tomography
IV: Intravenous
MRI: Magnetic resonance imaging
PCR: Polymerase chain reaction
